# circ‐AKT3 aggravates renal ischaemia‐reperfusion injury via regulating miR‐144‐5p /Wnt/β‐catenin pathway and oxidative stress

**DOI:** 10.1111/jcmm.16072

**Published:** 2020-11-16

**Authors:** Yong Xu, Wei Jiang, Lili Zhong, Hailun Li, Lin Bai, Xiaoling Chen, Yongtao Lin, Donghui Zheng

**Affiliations:** ^1^ Department of Nephrology Affiliated Huai’an Hospital of Xuzhou Medical University Huai’an, Jiangsu China; ^2^ Department of Nephrology Siyang Hospital of Traditional Chinese Medicine Suqian, Jiangsu China

**Keywords:** circ‐AKT3, circRNA, miR‐144‐5p, oxidative stress, renal ischaemia‐reperfusion injury, Wnt/β‐catenin signal

## Abstract

Renal ischaemia‐reperfusion (RI/R) injury is one major pathological state of acute kidney injury (AKI) with a mortality rate ranking 50% to 80%. MiR‐144‐5p acts as a molecular trigger in various diseases. We presumed that miR‐144‐5p might be involved RI/R injury progression. We found that RI/R injury decreased miR‐144‐5p expression in rat models. MiR‐144‐5p downregulation promoted cell apoptosis rate and activated Wnt/β‐catenin signal in RI/R injury rats. By performing bioinformatic analysis, RIP, RNA pull‐down, luciferase reporter experiments, we found that circ‐AKT3 sponged to miR‐144‐5p and decreased its expression in RI/R injury rats. Moreover, we found that circ‐AKT3 promoted cell apoptosis rate and activated Wnt/β‐catenin signal, and miR‐144‐5p mimic reversed the promotive effect of circ‐AKT3 in rat models. We also found that circ‐AKT3 increased the oxidative stress level in rat models. In conclusion, our study suggests that the circAKT3 is involved RI/R injury progression through regulating miR‐144‐5p/Wnt/β‐catenin pathway and oxidative stress.

## 1 INTRODUCTION

Renal ischaemia‐reperfusion (RI/R) injury is one majority pathology state of acute kidney injury (AKI) with a high mortality rate ranking 50%‐80%.^1^ The recovery of insufficient blood perfusion supplying to ischaemic organs leads to organ dysfunction and structural damage and causes RI/R injury.^2^, ^3^ Clinically, kidney transplantation,^4^ sepsis^5^ and cardiac surgery^6^ are the main causes of RI/R injury. In the past decades, the mechanisms of RI/R injury development have been continuously explored. However, the molecular triggers under RI/R injury occurrence and progression are still poorly understood.

MicroRNAs (miRNAs) are one type of non‐coding RNAs, which are a single strand consisting of 22‐24 nucleotides, and regulate gene‐expression at posttranscriptional level.^7^ MiRNAs research in the physiological and pathological progress of various diseases reveals its crucial roles in gene‐expression and biological progression.^8^, ^9^ Previous studies have elucidated that miRNAs are involved in the progression of RI/R injury. The protective effect of miR‐377 inhibitor in RI/R progression by inhibiting inflammation and oxidative stress.^10^ MiR‐424 alleviates RI/R injury through regulating p53 and death receptor 6 pathway.^11^ MicroRNA‐381 promotes RI/R injury progression by inhibiting CXCR4 expression.^12^ MiR‐155 is involved in the progression of RI/R injury by regulating FoxO3a.^13^ All the above studies suggest that miRNAs are essential for RI/R injury initiation and progression. Recently, with the innovation of technology, circular RNAs (circRNAs) have drawn widely scientific attention and have been in‐depth studied.^14^, ^15^ By acting as a competing endogenous RNA binding to miRNA and regulating its functions, the circRNA‐miRNA network has been revealed as an essential regulatory mechanism under disease progression.^16^ However, it is little known about the function of the circRNA‐miRNA network in RI/R injury progression.

MiR‐144‐5p, as a tumour suppressor, has been studied in bladder cancer,^17^ ovarian cancer,^18^ non‐small‐cell lung cancer^19^ and colorectal cancer.^20^ However, the role of miR‐144‐5p in the progression of RI/R injury is undocumented. Here, due to the crucial functions of miR‐144‐5p in variety of biological progressions,^21^, ^22^ we have been suggested that miR‐144‐5p might participate in RI/R injury progression in a circRNA‐miRNA network way.

In this study, by performing a series in *vivo* and *vitro* experiments, we identified the biological effects of miR‐144‐5p in RI/R injury progression. Furthermore, the investigation on the molecular mechanism of miR‐144‐5p revealed that circ‐AKT3 was a novel factor involving RI/R injury progression via regulating miR‐144‐5p and oxidative stress.

## 2 MATERIALS AND METHODS

### 2.1 Cell culture and cell ischaemia‐reperfusion model

Human renal epithelial cells HK‐2 and rat renal epithelial cells NRK‐52E were purchased from ATCC and cultured in a 90% DMEM/F12 + 10% FBS complete medium in a 37°C, 5% CO_2_ incubator. HK‐2 cells were routinely cultured in culture dishes. After the cells were confluent 80%, 3 mL of anoxic liquid saturated with high‐purity nitrogen for 30 minutes was added, and placed in a 37°C incubator with 94% N2, 1% O_2_ and 5% CO_2_. After 4 hours of hypoxia treatment, the oxygen treatment was carried out in a 37°C incubator with 5% CO_2_. This hypoxic reoxygenation model can well simulate IRI in vivo.

### 2.2 Transfection

The miR‐144‐5p mimic, inhibitor and its NC control were synthesized by GenePharma (Shanghai, China). Lipofectamine RNAiMAX (Life Technologies) was used to carry out all cell transfection approach following manufacture's protocol. The method for constructing circ‐AKT3 overexpressing in cells and rats was applied as previous report.^23^, ^24^ Briefly, in cells, the overexpression vector PcDNA‐AKT3 and its normal control vector were brought from GenePharma, and stably transfected into cells via lentivirus. Transfection efficiency was assessed by qRT‐PCR. In rats, AAV harbouring circ‐AKT3 vector was constructed and commercially obtained from Obio Technology. Collectively, AKT3 exon was inserted into PcDNA3.1 along with the endogenous flanking sequence. Upstream flanking sequence was partly copied and inserted in downstream in an inverted form. Adeno‐X™ Expression System (Clontech) was applied to construct adenovirus following manufacture's instruction. Sequences of miR‐144‐5p mimic, inhibitor, circ‐AKT3 vector were presented as following: miR‐144‐5p inhibitors 5′‐AGUACAUCAUCUAUACUGUA‐3′, miR‐144‐5p mimics 5′‐UACAGUAUAGAUGAUGUACU‐3′, circ‐AKT3 5′‐TTCGAATTCAGTGCTGAGATTACAGGCGTGAG‐3′,5′‐TTCGAATTCAGTGCTGAGATTACAGGCGTGAG‐3′.

### 2.3 Cell apoptosis detection

Annexin‐V APC/7‐AAD double staining: the cells were placed in a six‐well plate for routine culture. After the cells were attached, the corresponding drug‐containing medium was added according to the group setting, and a negative control group was established. After the cells were digested and washed, 500 μL of Binding Buffer was added to the suspension; 5 μL of Annexin‐V APC was added to mix, and 5 μL of 7‐AAD was then added to mix; the reaction was carried out for 10 minutes at room temperature in the dark, and the apoptosis was detected by FACS Cali bur flow cytometry (Becton‐Dickinson).

### 2.4 Construction of rat renal ischaemia‐reperfusion model

A total of forty‐five male Wistar rats (6‐week‐old, weighing 18‐22 g) were randomly divided into three groups, 15 in each group (experiment repeated three times), and fed for two weeks before the experiment. The operation of renal ischaemia/reperfusion injury was conducted as previously described.^25^ Before the operation, the rats were fasted overnight and anaesthetized with 20 g/L of pentobarbital sodium (40 mg/kg). The body temperature of rats was maintained at 36‐37°C during the operation. The rats were placed in the supine position, and the abdomen was selected under sterile conditions. For the rats of RI/R group, after the incision, the bilateral renal artery was bluntly separated, the right kidney was removed, and the left renal artery was clamped by a non‐invasive arterial clamp to establish a rat model of renal IR. After 45 minutes, the clamp was removed, the blood supply was restored and the kidney was gradually restored by dark red. The appearance of bright red blood indicates successful reperfusion. For the rats of the sham group, the bipartite renal pedicles were separated and covered with a piece of saline tissue. The right kidney was removed 45 minutes later. At last, the muscle layer and skin were sutured. The rats were placed in a 26‐28°C environment, and vital signs were observed until waking up. This operation was performed by two operators working together according to the recommendations in the Guide for the Care and Use of Laboratory Animals. All performance on the rat was approved by the Laboratory Animal Ethics Committee in Affiliated Huai'an Hospital of Xuzhou Medical University.

### 2.5 Renal function test

The whole blood was drawn and centrifuged at 3000 rpm for 10 minutes, and the supernatant was taken. According to the urea nitrogen determination kit and creatinine determination kit (GENECHEM, China), it is applied to automatic biochemical analyzer detection.

### 2.6 Oxidation measurement

The commercial kits of malondialdehyde (MDA), catalase (CAT), and superoxide dismutase (SOD) were purchased from Beyotime. O_2_^‐^ assay kit was obtained from Jiancheng Bioengineering. The kidney tissues were harvested after one day of I/R surgery accomplished. The levels of MDA, O_2_^‐^, CAT and SOD were measured by commercial kits, respectively, following manufacture's protocol. Experiments were repeated three times.

### 2.7 *Fluorescent* in situ *hybridization (FISH)*

The FISH assay was conducted in HK‐2 cells following previous study.^23^ The biotin‐labelled circ‐AKT3 probe was purchased from Genepharma. Collectively, Cy5‐conjugated streptavidin (Life Technologies) was applied to detect biotin‐labelled circ‐AKT3 in HK‐2 cells. And nuclei were stained with DAPI. Lecia TCS SP2 AOBS (Lecia) microscope was used (400x magnification).

### 2.8 HE staining detection

Pretreatment paraffin section: dewaxing according to the conventional method, hydration, soaking the slices with xylene for 5 minutes, replacing xylene and then soaking for 5 minutes; soaking in absolute ethanol for 5 minutes; soaking in 95% ethanol for 5 minutes; 85% ethanol soak for 5 minutes; soak for 5 minutes in 70% ethanol, immerse in PBS for 3 minutes × 3 times. The experimental procedure is based on the protocol of the Hematoxylin‐Eosin staining kit (GENECHEM).

### 2.9 TUNEL method for apoptosis detection

Dewaxing and hydration steps were carried out according to a conventional method, and the sections were immersed in xylene for 5 minutes, and then replaced with xylene and then immersed for 5 minutes. Then use different concentrations of ethanol for soaking. The apoptosis detection step is based on the protocol of the TUNEL detection apoptosis kit (GENECHEM).

### 2.10 Transmission electron microscopy (TEM) observation of subcellular structure

The tissue was excised, washed once with PBS and placed in an EP tube. After the fixation, dehydration, embedding and solidification procedures were carried out in accordance with the conventional procedures, the LKB‐1 ultrathin slicer was used for sectioning (50‐60 nm). After double staining with 3% uranyl acetate‐lead lead, the photograph was taken with a JEM‐1400 transmission electron microscope (JEOL).

### 2.11 Western blot

Protein was extracted from the cells using RIPA (radioimmunoprecipitation) lysis buffer, then fractionated on 10% sodium dodecyl sulphate‐polyacrylamide gel electrophoresis (SDS‐PAGE) and transferred to a polyvinylidene fluoride (PVDF) membrane by electroblotting. After blocking for 1.5 hours at room temperature with shaking, the membrane was incubated with the target protein primary antibody at 4°C overnights. The membrane was then incubated with the secondary antibody for 1 hour at room temperature with a 1:2000 dilution. SYNGENEG: BOX chemiXR5 software was applied to detect the intensity of the visual signals. Antibody used in this experiment as following: Bax (abcam; ab32503), Bcl‐2 (abcam; ab196495), c‐caspase3, (abcam; ab4051), β‐catenin (abcam; ab32572), c‐myc (CST; 18 583), cyclinD1 (CST; 2978), GAPDH (CST; 5174), β‐actin (CST; 4970).

### 2.12 Quantitative reverse transcription polymerase chain reaction

Total RNA was isolated using TRIzol reagent (Invitrogen, Carlsbad, CA, USA) and then reverse transcribed using the RevertAid First Strand cDNA Synthesis kit (Thermo Fisher Scientific). The qRT‐PCR analysis was further carried out in ABI Step one plus Real‐time PCR system (Applied Biosystems) using Real‐time PCR Master Mix (SYBR Green) (TOYOBO). The relative expression levels of the target genes were normalized to the relative expression levels of the endogenous control (GAPDH) by using the 2^‐ΔΔCt^ method. PcDNA‐AKT3 and its NC plasmids were constructed following previous report.^26^ Primers used in this experiment as following;

**Table:** 

Gene	Forward 5'~3'	Reverse 3'~5'
Bax	CACCAGCTCTGAACAGATC	CTTCTTCCAGATGGTGAGC
Bcl‐2	CCTGAGAGCAACCGAACG	CCTGAGAGCAACCGAACG
c‐caspase3	GTGGAACTGACGATGATATGGC	CGCAAAGTGACTGGATGAACC
β‐catenin	ATGACTCGAGCTCAGAGGGT	ATTGCACGTGTGGCAAGTTC
c‐myc	GGCTCCTGGCAAAAGGTCA	CTGCGTAGTTGTGCTGATGT
cyclinD1	CGAGGAGCTGCTGCAAATGG	CAGAGGGCAACGAAGGTCTG
GAPDH	GGAGCGAGATCCCTCCAAAAT	GGCTGTTGTCATACTTCTCATGG
miR‐144‐5p	CGGGCGATATCATCATATACTG	GTGCAGGGTCCGAGGT
circ‐AKT3	TGGTTCGAGAGAAGGCAAGTG	CTGTCCATTCTTCTCTTTGCGA

### 2.13 Dual‐luciferase assay

The wild type and mutant type of circ‐AKT3 3’‐UTR, and miR‐144‐5p binding sites were generated. PmirGLO dual‐luciferase reporter plasmids (Genechem) were consisting of circ‐AKT3 3‐UTR, WT and Mut. Relative luciferase intensity was detected by Dual‐luciferase reporter assay Kit (Promega) according to the manufacturer instructions.

### 2.14 RNA pull‐down

Bio‐circ‐AKT3‐WT, Bio‐circ‐AKT3‐Mut and its negative control generated at Genechem company. The above‐biotinylated probes were transfected into 293T cells for 2 days. The cell lysates, treated with circ‐AKT3‐RNA beads, washed for 5 times. PCR assay was used to analysis the bond RNA.

### 2.15 RNA‐binding protein immunoprecipitation assay

293T cells were transfected with miR‐144‐5p mimics and Myc‐AGO2. AGO2 protein immunoprecipitation assay was conducted after 2 days. The treated 293T cells were lysed by using RNA immunoprecipitation (RIP) buffer, in a designated buffer. Next, the IP's and input were handed to 1 mL Trizol isolating RNA. The eluted RNA was carried out by synthesizing cDNA for qRT‐PCR to detect the binding targets of miR‐144‐5p.

### 2.16 Statistical analysis

The SPSS 17.0 (SPSS Inc) was used to perform statistical analysis. The variance (ANOVA) or Student's t test was performed to analyse the comparisons among different groups. The Mean ± SD was conducted to present experimental results. Differences with *P* < .05 were considered to be significantly different among groups.

## 3 RESULTS

### 3.1 MiR‐144‐5p inhibitor promotes RI/R injury progression

**Figure 1:**
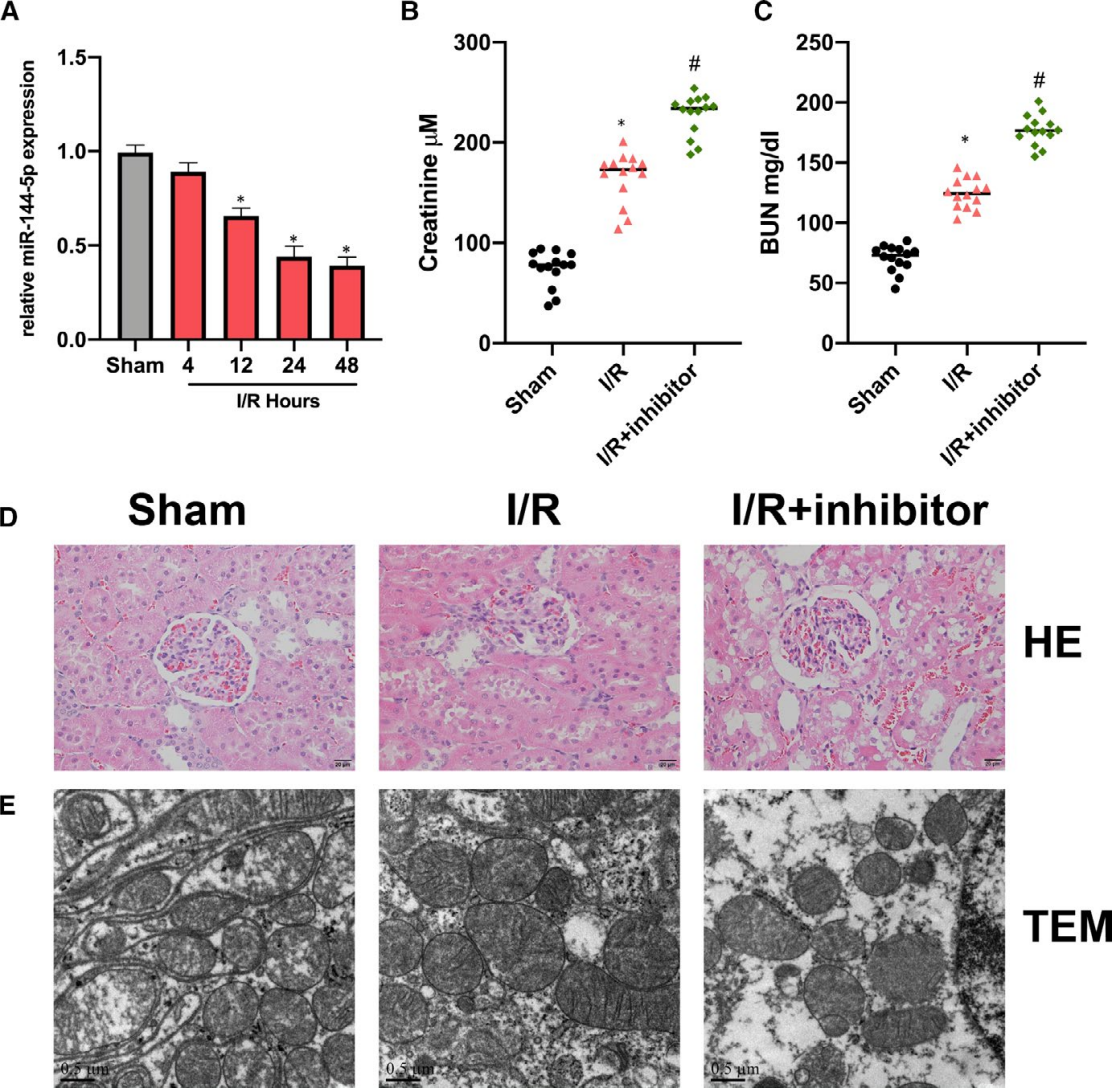
MiR‐144‐5p inhibitor promotes RI/R injury progression A, qRT‐PCR was performed to evaluate the expression of miR‐144‐5p RI/R rat and sham rat kidney tissues at postoperative time 4, 12, 24, 48 h. B‐C, Tail vein injection was conducted to inject miR‐144‐5p inhibitor and its NC plasmids into the rats, and then the levels of BUN and Serum Cr were evaluated by commercial kit. D, Observation of miR‐144‐5p inhibitor effect on RI/R injury rats presented by HE staining. E, Observation of cellular ultrastructure changes of miR‐144‐5p inhibitor on RI/R injury rats by TEM. N = 6 in each group. Experiments were repeated three times. *P < .05, ^#^ *P* < .05

First, we found that miR‐144‐5p expression in RI/R rat kidney tissues was decreased in comparison with the sham group. (Figure 1A). Next, we found miR‐144‐5p inhibitor up‐regulated BUN and Serum Cr in RI/R rats (Figure 1B, C). Then, HE staining results indicated that the renal tubular epithelial cells in RI/R rats showed significant oedema and necrosis. With the additional miR‐144‐5p inhibitor injection, the cell damage degree was elevated (Figure 1D). TEM images showed the cell damage in the miR‐144‐5p inhibitor group was significantly promoted (Figure 1E).

### 3.2 MiR‐144‐5p inhibitor promotes cell apoptosis and activates Wnt/β‐catenin signal

**Figure 2:**
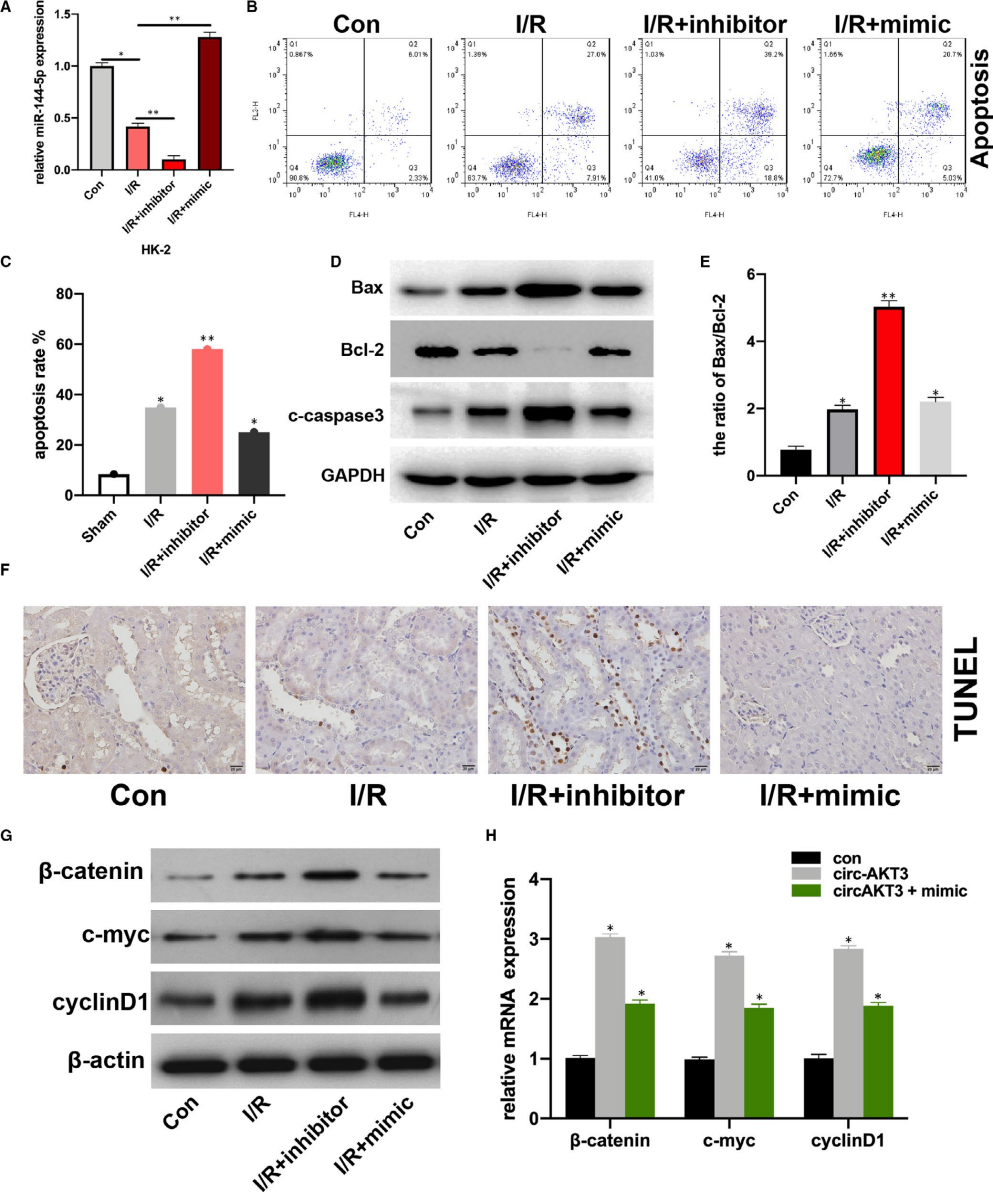
MiR‐144‐5p inhibitor promotes cell apoptosis and activates Wnt/β‐catenin signal A, qRT‐PCR assays were applied to evaluate the transfection efficiencies of miR‐144‐5p mimic, inhibitor and its NC plasmids in hypoxia treated HK‐2 cells. B, Apoptosis rate of HK‐2 cells (con), hypoxia‐treated HK‐2 cells (I/R), miR‐144‐5p inhibitor transfected hypoxia‐treated HK‐2 cells (I/R + inhibitor) and miR‐144‐5p mimic transfected hypoxia‐treated HK‐2 cells (I/R + mimic) was measured by flow cytometry. C, Flow cytometry data were calculated and showed. D, Apoptosis protein Bax, Bcl‐2 and c‐caspase3 in cell models were measured by Western blot. E, Bax/Bcl‐2 ratio was showed. F, Apoptotic cells in miR‐144‐5p inhibitor/mimic and its NC‐treated RI/R injury rat kidney tissues by were detected by TUNEL method. G and H: the β‐catenin, c‐myc and cyclinD1 level in treated RI/R rat kidney tissues was measured by Western blot (G) and qRT‐PCR (H). Each experiment was performed three times, *P < .05, **P < .01

To investigate the role of miR‐144‐5p in renal I/R progression, firstly, we transfected miR‐144‐5p mimic, inhibitor and its NC plasmids into hypoxia‐treated HK‐2 cells, transfection efficiencies were measured (Figure 1A). Then, we detected the apoptotic rate of HK‐2 cells (con), hypoxia‐treated HK‐2 cells (I/R), miR‐144‐5p inhibitor‐transfected I/R HK‐2 cells (I/R + inhibitor), and 144‐5p mimic‐transfected I/R HK‐2 cells (IR + mimic), respectively. We found that the cell apoptosis rate was significantly increased in I/R + inhibitor group in comparison with I/R group, and miR‐144‐5p mimic decreased cell apoptosis rate in I/R + inhibitor group compared with I/R group (Figure 2B, C). That phenomenon was coupled with up‐regulated expression of Bax and c‐caspase3, and down‐regulated expression of Bcl‐2 in the I/R + inhibitor group compared with I/R group (Figure 2D). The ratio of Bax/Bcl‐2 in I/R + inhibitor group was significantly higher than I/R group (Figure 2E). By performing TUNEL assay, we found the percentage of apoptosis cells was significantly up‐regulated in the I/R rats with the additional miR‐144‐5p inhibitor injection (Figure 2F). The above results suggested that down‐regulated miR‐144‐5p promoted cell apoptosis in upon I/R condition. Furthermore, the expression of β‐catenin, c‐myc, and cyclinD1 in RI/R rat kidney tissues was increased by miR‐144‐5p inhibitor, while reversed by miR‐144‐5p mimic (Figure 2G, H). This result coupled with a interesting phenomenon, which is miR‐144‐5p mediated the expression of β‐catenin, c‐myc and cyclinD1 both in mRNA and protein level upon renal I/R condition.

### 3.3 Circ‐AKT3 sponges to miR‐144‐5p

**Figure 3:**
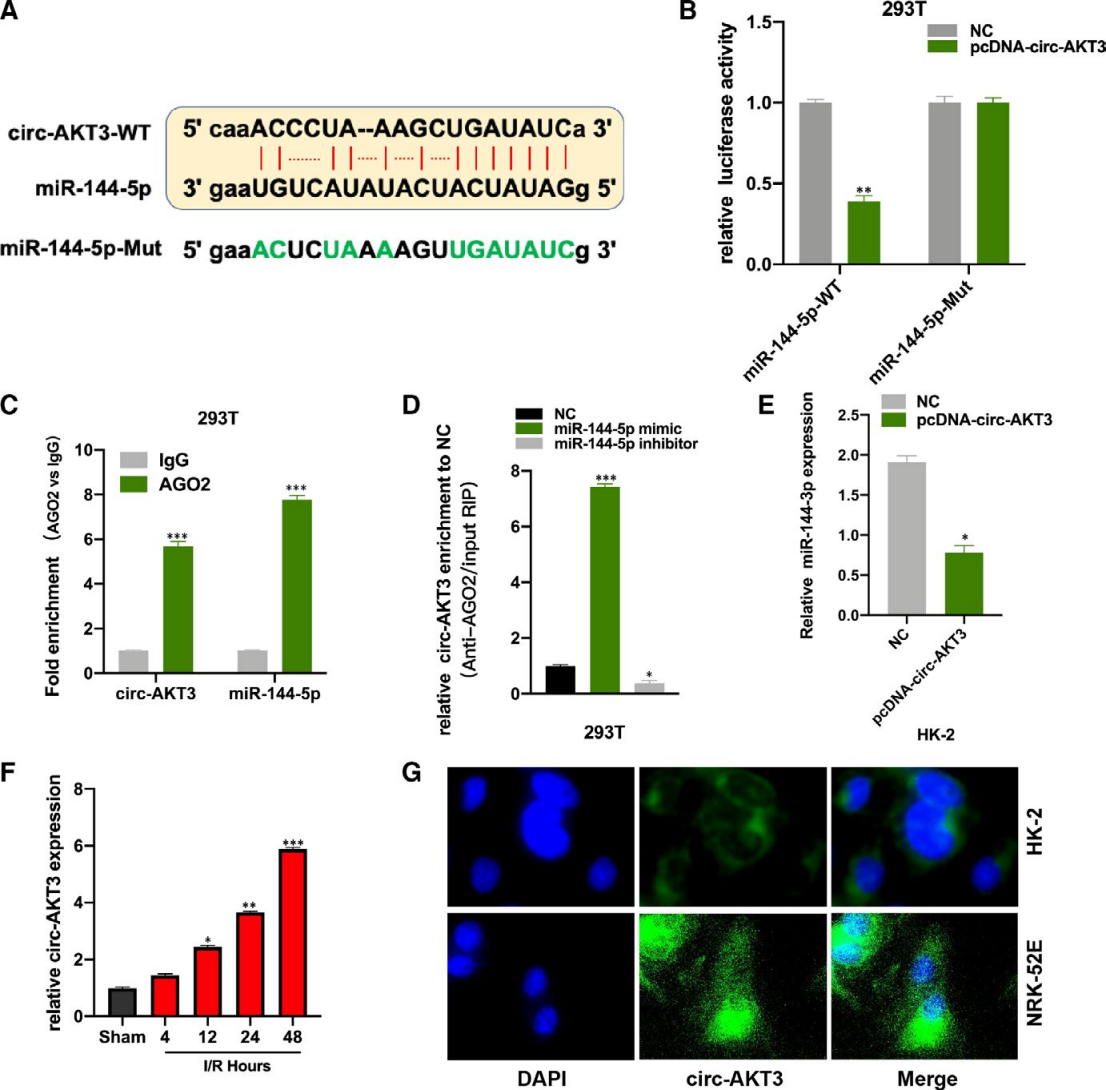
Circ‐AKT3 sponges to miR‐144‐5p A, binding sites between circ‐AKT3 and miR‐144‐5p. B, The luciferase activity in 293T cells was measured by Dual‐luciferase assay. C, The correlation between miR‐144‐5p and circ‐AKT3 in 293T cells was tested by using RIP assay, anti‐Ago2 and anti‐IgG antibody were used to immunoprecipitated cellular lysates. D, The relationship between miR‐144‐5p and circ‐AKT3 in 293T cells was tested by using RNA pull‐down experiment. E, relative expression of miR‐144‐5p in pcDNA‐circ‐AKT3 and its NC plasmids treated rat models were measured by qRT‐PCR. F, circ‐AKT3 expression in RI/R rat and sham rat kidney tissues at postoperative time 4, 12, 24, 48 h were measured by qRT‐PCR. G, the representative images of FISH of circ‐AKT3 expression in HK‐2 and NRK‐52E cells. Three independent experiments were carried out. *P < .05, **P < .01, ***P < .001

By performing the bioinformatic analysis (http://starbase.sysu.edu.cn/index.php), we found that circRNA AKT3 (circ‐AKT3) is a promising sponge to miR‐144‐5p, and the binding sites between miR‐144‐5p and circ‐AKT3‐WT were showed (Figure 3A). The PcDNA‐circ‐AKT3 and its NC plasmids were respectively co‐transfected with the luciferase vector harbouring miR‐144‐5p WT and Mut sequence into 293T cells. Luciferase activity in miR‐144‐5p WT and PcDNA‐circ‐AKT3 plasmids co‐transfected 293T cells was significantly decreased (Figure 3B). The AGO2‐RIP assay results showed that circ‐AKT3 and miR‐144‐5p were abundantly enriched in the anti‐AGO2 pellet (Figure 3C). It was found that circ‐AKT3 was more enriched in the miR‐144‐5p mimic group in comparison with the NC group in RNA pull‐down experiment (Figure 3D). And we found circ‐AKT3 decreased miR‐144‐5p expression in HK‐2 cells (Figure 3E). Moreover, we measured circ‐AKT3 expression in RI/R injury rat kidney tissues, and circ‐AKT expression was increased in a time‐dependent manner (Figure 3F). We also detected circ‐AKT3 in HK‐2 and NRK‐52E cells by FISH, and we found that circ‐AKT3 mainly distributed in cytoplasmic (Figure 3G).

### 3.4 Circ‐AKT3 promotes RI/R injury progression by sponging to miR‐144‐5p

**Figure 4:**
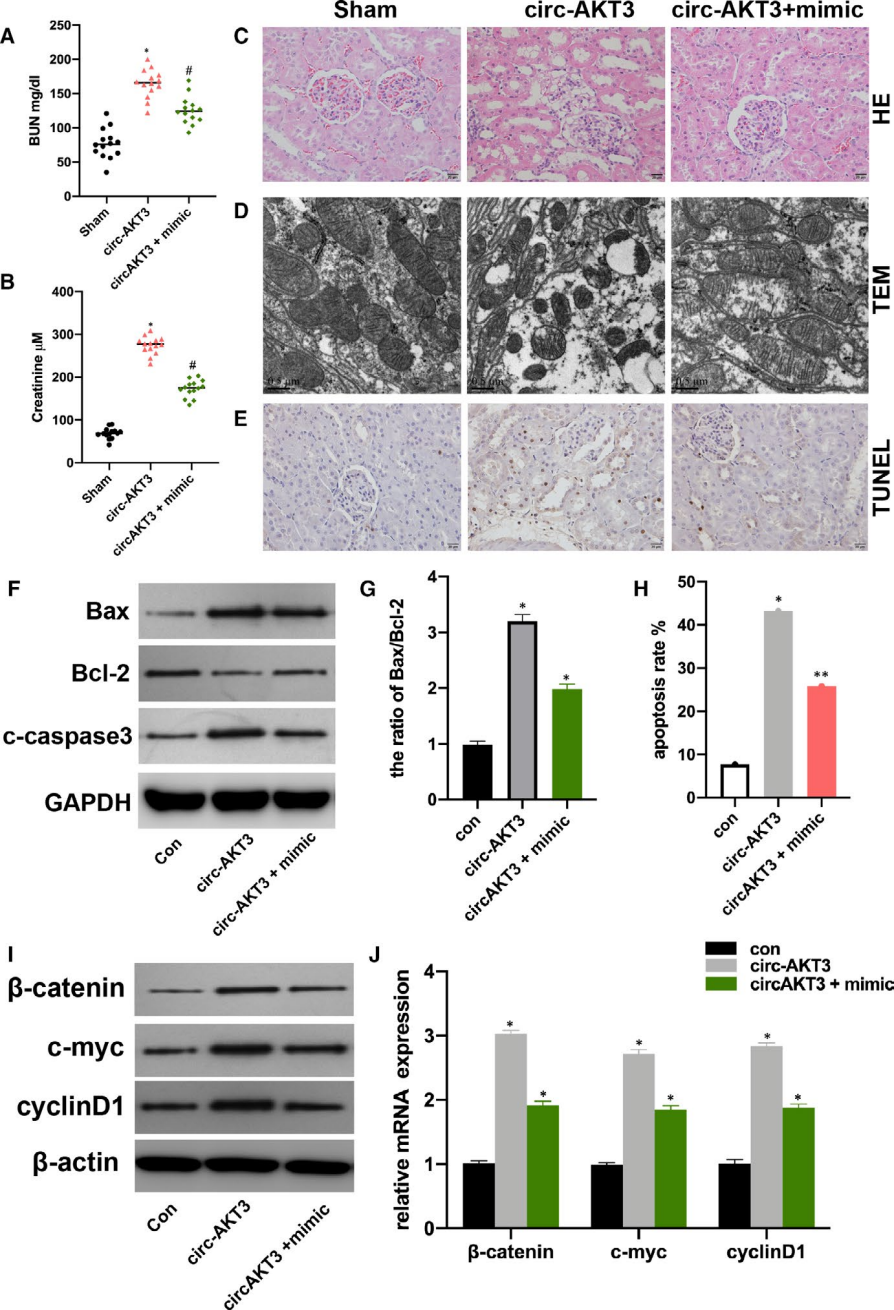
Circ‐AKT3 promotes RI/R injury progression by sponging to miR‐144‐5p A and B: Tail vein injection was conducted to inject pcDNA‐circ‐AKT3, pcDNA‐circ‐AKT3 + miR‐144‐5p mimic plasmids into the RI/R injury rats, and the level of BUN and Serum Cr were measured by qRT‐PCR. C, Observation of the effect of pcDNA‐circ‐AKT3, pcDNA‐circ‐AKT3 + miR‐144‐5p mimic on RI/R injury rats presented by HE staining. D, Observation of cellular ultrastructure changes of pcDNA‐circ‐AKT3, pcDNA‐circ‐AKT3 + miR‐144‐5p mimic on RI/R injury rat by TEM. E, Detection of the effect of pcDNA‐circ‐AKT3, pcDNA‐circ‐AKT3 + miR‐144‐5p mimic on apoptotic ratio in renal I/R injury tissues by TUNEL method. F, the expression of Bax, Bcl‐2 and c‐caspase3 in pcDNA‐circ‐AKT3, pcDNA‐circ‐AKT3 + miR‐144‐5p mimic transfected hypoxia‐treated HK‐2 cells were evaluated by Western blot. G, Bax/Bcl‐2 ratio was shown. H, Apoptosis rate of HK‐2 cells (con), hypoxia‐treated HK‐2 cells (circ‐AKT3), and pcDNA‐circ‐AKT3 + miR‐144‐5p mimic transfected hypoxia‐treated HK‐2 cells (circ‐AKT3 + mimic) was measured by flow cytometry. G, the level of β‐catenin, c‐myc and cyclinD1 in pcDNA‐circ‐AKT3, pcDNA‐circ‐AKT3 + miR‐144‐5p mimic transfected RI/R rat kidney tissues was measured by Western blot (I) and qRT‐PCR (J). Each experiment was repeated three times, *P < .05, ^#^ *P* < .05, **P < .01

The above results suggested that circ‐AKT3 promoted RI/R injury progression by targeting miR‐144‐5p. At first, we measured the BUN and serum Cr level in sham, pcDNA‐circ‐AKT3 + miR‐144‐5p mimic and NC plasmids injected rat models. Circ‐AKT3 promoted BUN and serum Cr level and miR‐144‐5p reversed the circ‐AKT3 promotive effect (Figure 4A, B). The HE staining, TEM, and TUNEL assay results showed that pcDNA‐circ‐AKT3 promoted cell damage and cell apoptosis, and miR‐144‐5p mimic reversed it (Figure 4C‐E). Next, we found that circ‐AKT3 promoted cell apoptosis level and it was alleviated by miR‐144‐5p mimic (Figure 4F‐G). Moreover, it was found that circ‐AKT‐3 increased Wnt/β‐catenin signal protein level, and miR‐144‐5p extenuated the effect of circ‐AKT3.

### 3.5 Circ‐AKT3 enhances RI/R injury‐induced oxidative stress

**Figure 5:**
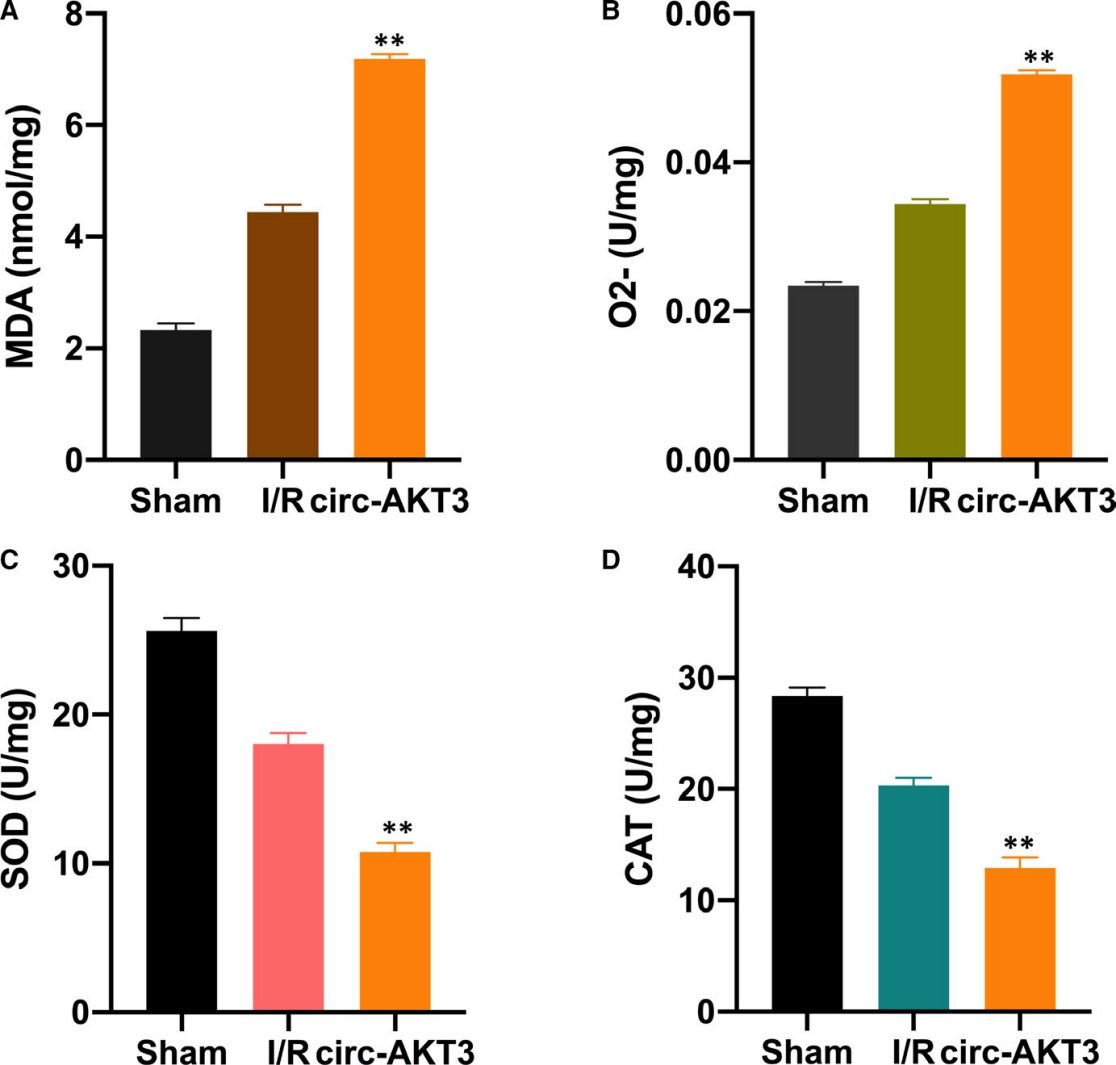
Circ‐AKT3 enhances RI/R injury‐induced oxidative stress pcDNA‐circ‐AKT3 (AAV) and its NC were injected into rat by performing tail vein injection surgery. After 24 h, tissues were collected for MDA (A), O_2_^‐^ (B), SOD (C), CAT (D) measurement. N = 6 in each experiment. **P < .01

Here, we explored the effect of circ‐AKT3 on RI/R‐induced oxidative stress. MDA is one of the byproducts of lipid peroxidation. SOD and CAT are enzymes that clear ROS away..^10^, ^27^ We investigated the level of MDA, O_2_^‐^, SOD, and CAT in the sham, RI/R and RI/R + circ‐AKT3 rat group, respectively. In comparison with the I/R group, the MDA and O_2_ levels were significantly increased in the RI/R + circ‐AKT3 group (Figure 5A, B). Moreover, the SOD and CAT levels were significantly down‐regulated in the I/R + cric‐AKT3 group (Figure 5C, D).

## 4 DISCUSSION

RI/R injury, with a high mortality rate and various high‐risk factors, has been a global health burden. Due to its inefficiency in clinical management and lacking effective medications, it is imperative to find promising therapeutic targets. Here, by performing a series in vivo and vitro experiments, our findings suggest that circ‐AKT3 promotes RI/R injury progression via sponging to miR‐144‐5p and regulating oxidative stress.

First, we investigated the role of miR‐144‐5p in the RI/R injury progression. We found that RI/R injury decreased miR‐144‐5p expression. By evaluating BUN and serum Cr level in rat models, we found that miR‐144‐5p down‐regulation promoted RI/R injury progression. In order to investigate the biological function of miR‐144‐5p, we conducted cell apoptosis assay, TUNEL and TEM assay. The role of cell apoptosis in RI/R injury progression has been demonstrated in many studies.^28^, ^29^, ^30^, ^31^ It was found that miR‐144‐5p down‐regulation promoted cell apoptosis rate and elevated cell damage degree. The previous study has elucidated that Wnt/β‐catenin signal participates in the RI/R injury progression..^32^ Here, we tested the effect of miR‐144‐5p on Wnt/β‐catenin signal in rat models; we found that miR‐144‐5p down‐regulation activated Wnt/β‐catenin signal, indicating that miR‐144‐5p modulates RI/R injury progression via Wnt/β‐catenin signal. Subsequently, its underlying molecular mechanism was investigated. By performing bioinformatic analysis, we found that circ‐AKT3 was a potential sponge to miR‐144‐5p.

CircRNAs are involved in various cellular events, and the circRNA‐miRNA network is its main molecular pattern.^33^ Circ‐AKT3 has been found as a tumour suppressor in the development of glioblastoma^34^ and renal clear cell carcinoma.^26^ We speculated that circ‐AKT3 might be involved in RI/R injury progression via sponging to miR‐144‐5p. The luciferase reporter, AGO2‐RIP and RNA pull‐down experiments were performed. We found that circ‐AKT3 sponged to miR‐144‐5p and regulated its expression in rat models. Moreover, it was found that circ‐AKT3 promoted cell apoptosis rate and activated Wnt/β‐catenin signal, while reversed by miR‐144‐5p. Moreover, our results showed that circ‐AKT3 increased MDA and O_2_ level, and down‐regulated SOD and CAT enzymes level in RI/R injury rats, it indicated that circ‐AKT3 promoted oxidative stress level.

In the current study, our findings suggested that circ‐AKT3 participated in RI/R injury through regulating Wnt/β‐catenin signal via sponging to miR‐144‐5p. While, the association between miR‐144‐5p and Wnt/β‐catenin signal in RI/R injury progression remains unclear for now. Due to the molecular character of microRNA, we are investigating its downstream mRNA target and explore its correlation with Wnt/β‐catenin signal. Furthermore, our results showed that circ‐AKT3 mediates oxidative stress level in RI/R rats but its underlying mechanism demands further exploration in our further study.

Collectively, all the above results indicate that circ‐AKT3 sponges to miR‐144‐5p and regulates its expression. And we identified the promotive effect of circ‐AKT3 in the progression of RI/R injury. Our study provides a promising therapeutic target for RI/R injury management and a new insight for RI/R injury basic research.

## CONFLICT OF INTEREST

The authors confirm that there are no conflicts of interest.

## AUTHOR CONTRIBUTION

Yong Xu: Conceptualization (equal); Data curation (equal); Formal analysis (equal); Investigation (lead); Methodology (equal); Project administration (equal); Writing‐original draft (equal). Wei Jiang: Conceptualization (equal); Data curation (equal); Formal analysis (equal); Investigation (equal); Software (equal); Visualization (equal). Lili Zhong: Data curation (equal); Formal analysis (equal); Investigation (equal); Methodology (equal); Visualization (equal). Hailun Li: Software (equal); Validation (equal); Visualization (equal). Lin Bai: Data curation (equal); Software (equal); Validation (equal); Visualization (equal). Xiaoling Chen: Data curation (equal); Software (equal); Validation (equal). Yongtao Lin: Data curation (equal); Investigation (equal); Visualization (equal). Donghui Zheng: Conceptualization (lead); Investigation (lead); Methodology (lead); Project administration (lead); Resources (lead); Supervision (lead); Writing‐original draft (lead); Writing‐review & editing (lead).

## Data Availability

The data sets used and/or analysed during the current study are available from the corresponding author on reasonable request.
